# Reporting heterogeneity in the associations between personality and health problems: Anchoring self-reports with health vignettes

**DOI:** 10.1177/13591053241285960

**Published:** 2024-10-06

**Authors:** Markus Jokela

**Affiliations:** University of Helsinki, Finland

**Keywords:** bias, health, personality, self-report, vignette

## Abstract

Associations between personality and self-reported health problems may be biased by reporting heterogeneity, that is, tendency to rate the severity of the same health problem differently. This study used hypothetical health vignettes to examine the magnitude of such heterogeneity. Participants were from Health and Retirement Study (HRS; *n* = 3950; mean age 65 years, range from 30 to 97) and Wisconsin Longitudinal Study (WLS; *n* = 8664; mean age 64 years, range from 34 to 87). Personality traits of the Five Factor Model (extraversion, emotional stability, agreeableness, conscientiousness, and openness to experience) were only weakly associated with vignette ratings (*r*s < 0.10). Associations between personality and self-reported health problems were not substantially changed when the thresholds of self-reported severity were allowed to vary by personality, based on the participants’ ratings of the vignettes. Reporting heterogeneity does not appear to be a major source of bias in the associations between personality traits and self-reported health problems.

## Introduction

Health is partly determined by personality. Personality traits, low conscientiousness in particular, have been associated with adverse health outcomes, such as mortality risk (Graham et al., 2017; Jokela et al., 2020), depressive symptoms (Hakulinen et al., 2015), and functional disability (Jokela, 2018). Personality can also change in response to changes in health status, such as development of chronic diseases (Jackson et al. 2017; Jokela et al., 2014). Some of the health associations can be demonstrated with objective measures of health, including biomarkers (e.g. inflammation; Graham et al., 2018), physical measures (e.g. grip strength or walking speed; Stephan et al., 2017), or mortality status (Jokela et al., 2020).

Other health measures rely on self-reported data that may be confounded by differences in how people rate health problems. Individuals with low emotional stability, for instance, might rate their sleep problems, chronic pain, depressive symptoms, or functional limitations more severe than individuals with high emotional stability, even if their objective health status were the same (Feldman et al., 1999). One study showed that the longitudinal associations between personality and health were different for objective and subjective measures of health (Wettstein et al., 2017). Similar discrepancies between subjective and objective health correlates of personality traits have been reported (Costa and McCrae, 1980, 1987; Kööts-Ausmees et al., 2016), but these studies have not offered solutions to these possible problems with self-rated health indicators.

Health vignettes have been proposed as one solution to quantify and mitigate the problem of *reporting heterogeneity*, that is, individual differences between people in their tendency to differently rate the severity of a given health condition (Grol-Prokopczyk et al., 2015; King et al., 2004). Health vignettes can be used to anchor response scales by asking the participants to rate the severity of health problems of hypothetical characters described in short vignettes. For example, a health vignette for depressive symptoms might read:

Maria feels nervous and anxious. She worries and thinks negatively about the future, but feels better in the company of people or when doing something that really interests her. When she is alone, she tends to feel useless and empty. Overall, in the last 30 days, how much of a problem did Maria have with feeling sad, low, or depressed?

By asking the participants to rate multiple fictional health vignettes it is possible to evaluate whether personality traits correlate with people’s severity ratings of identical fictional health problems. This provides information on how individuals set their thresholds in rating the same health condition, say, as “mild” versus “moderate” or “moderate” versus “severe.” It is then possible to allow the severity thresholds of self-reported health scales to vary as a function of personality when estimating the associations between personality traits and self-rated health (King et al., 2004). For example, individuals with low emotional stability might have their severity thresholds set very low, so that even minor health problems would be rated more severe than by individuals with high emotional stability. By adjusting the severity thresholds to be equal across the personality trait continuum—based on the vignette ratings—it is possible to assess the association between personality and health outcome with less reporting heterogeneity.

The concept of reporting heterogeneity is close to the concept of response style which refers to use of the response scale that is unrelated to the actual content of the questions (Van Vaerenbergh and Thomas, 2013). Various response styles, such as extreme or acquiescence responding (using or not using the extreme ends of the scales, respectively) have been associated with personality differences. Higher openness to experience and conscientiousness, in particular, have been associated with both extreme and acquiescence responding, but higher extraversion, agreeableness, and emotional stability also correlate with more extreme response style (He et al., 2014; Hibbing et al., 2019). The reporting heterogeneity we are interested here is different from the concept of response style in that reporting heterogeneity is tied to the content of the question, so that some individuals rate the same health problem as more severe than others (King et al., 2004). However, the previously reported associations between personality and response styles provide additional motivation for the study of reporting heterogeneity, because they show that personality differences can influence how people respond to survey questions.

Health vignettes have been used particularly in studies of cross-cultural differences and health inequalities (Datta Gupta et al., 2010) and in studies comparing groups with different educational levels, ethnic backgrounds, or genders (Dowd and Todd, 2011). However, the method has not been applied in research on personality and health. The present study examined whether personality traits of the Five Factor Model were associated with severity ratings of health vignettes, and how much of the associations between personality and self-rated health problems could be explained by reporting heterogeneity. I hypothesized that lower emotional stability would be related to lower severity thresholds, because individuals with low emotional stability tend to be more sensitive to threats and negative emotions, and that highly conscientious individuals would have lower thresholds for vignette severity, because they may set higher standards for good health. There were no hypotheses for the other personality traits or between different domains of health problems. Data from two cohort studies were used to test whether the conclusions replicated across independent samples.

## Methods

### Participants and measures

The Health and Retirement Study (HRS) is a nationally representative longitudinal study of more than 30,000 individuals representing the U.S. population older than 50 years. Telephone or in-person interviews have been conducted every 2 years since baseline in 1992. Personality was assessed using a self-reported instrument that was adapted from the Midlife in the United States (MIDUS) study, with five items for extraversion (Cronbach alpha reliability = 0.74), four items for emotional stability (0.63), five items for agreeableness (0.78), five items for conscientiousness (0.63), and seven items for openness to experience (0.79), rated on a 4-point rating scale. The personality instrument was administered to half of the sample in 2006 and to the other half in 2008. Mean scores for personality scales were calculated for individuals with no more than one missing item in the scale. For the health vignettes, data were collected in a separate 2007 mail questionnaire which was sent to participants who had completed a self-interview in 2006 and who were not in two of the other additional data collection modules of 2007. Of the 5678 mailed questionnaires, 4639 were returned (response rate 82%). The participants were asked to rate their own health in six domains (feeling sad, low, or depressed; difficulty with sleeping; difficulty with concentrating or remembering things; problem with moving around; shortness of breath; pain or bodily aches) using a 5-point rating scale (1 = None, 2 = Mild, 3 = Moderate, 4 = Severe, 5 = Extreme). They then rated 18 corresponding health vignettes of fictional characters (three for each of the health domains with different levels of severity described in each of the three vignettes) using the same rating scale as with the self-rated health items. There were two versions of the health vignette questionnaires, which differed by the genders of the vignette characters and the order in which the vignettes were presented. Given that many of the participants were no longer in the workforce (53% reported not working for pay in 2006/2008), the item concerning limitations in the participant’s work (“Do you have any impairment or health problem that limits the kind or amount of work you can do?”) was left out of the analysis. For the sum scores, the Cronbach alpha reliability was 0.61 for depression, 0.88 for sleep, 0.54 for memory, 0.62 for movement, 0.70 for shortness of breath, and 0.51 for pain.

The Wisconsin Longitudinal Study (WLS) has followed a random sample of 10,317 Wisconsin high schools of 1957 who were born between 1937 and 1940 (5326 women, 4991 men). The present study used data from the 2003 to 2007 follow-up in which the vignette questionnaires were administered. In addition to the main sample, the WLS has also collected data siblings of the graduates (no more than one sibling per graduate). The data collection has been very similar for the siblings and graduates. In the present study, the sibling sample was combined with the graduate sample (*n* = 5582 graduates and 3082 siblings). Personality was assessed with a 29-version of the Big Five Inventory (BFI), rated on a 6-point response scale. The Cronbach alpha reliabilities were 0.76 for extraversion in graduates for extraversion, 0.78 for emotional stability, 0.69 for agreeableness, 0.64 for conscientiousness, and 0.61 for openness to experience. For the health vignettes, the participants were asked to rate their own health with four items (feeling sad, low, or depressed; problem with worry or anxiety; problems with moving around; difficulty with vigorous activities, such as running 2 miles or cycling) using a 5-point response scale (1 = None, 2 = Mild, 3 = Moderate, 4 = Severe, 5 = Extreme). There was a total of four vignettes of fictional characters for physical health and four for mental health, and each participant was randomly administered two of the physical-health vignettes and two of the mental-health vignettes, each of which was rated with two questions (sadness/depression and worry/anxiety for mental health; mobility and vigorous activities for physical health). For the sum scores, the Cronbach alpha reliability was 0.62 for depression, 0.56 for anxiety/worry, 0.59 for movement, and 0.73 for vigorous activities.

In both studies, the analytic sample was determined to be the participants who had data on personality and the health vignettes (*n* = 3950 for HRS, mean age 65 years, range from 50 to 97 years; *n* = 8664 for WLS, mean age 64 years, range from 34 to 87 years), all the other participants were excluded from this analysis. Both datasets are available to download from their websites (HRS: https://hrsonline.isr.umich.edu; WLS: https://www.ssc.wisc.edu/wlsresearch/). The exact wording of the vignettes can be found in the Supplemental Material.

In the WLS, data was initially collected via a telephone interview, after which a questionnaire was mailed to the participants. Verbal informed consent was obtained at the beginning of the telephone interview. All instruments and operations were approved by the Institutional Review Board of the University of Wisconsin-Madison. In the HRS, all participants provided written informed consent, and HRS data collection was approved by the Institutional Review Board at the University of Michigan. According to the guidelines of the Finnish National Advisory Board on Research Integrity (https://tenk.fi/en), a research project that uses only de-identified public or archived data does not require an Institutional Review Board approval, and thus no IRB approval was required for this particular study.

### Statistical analysis

The associations of personality with self-rated health and health vignettes were analyzed using ordered probit regression that can be used to study variables with ordered categorical response scales (King et al., 2004). The probability of observing the outcome *i* is modeled as: Pr(oj = i) = Pr(κi−1 < β1x1j + β2x2j +· · · + βkxkj + uj ≤ κi), where β denotes regression coefficients, x denotes predictor variable, u denotes error term, and κ denotes threshold cutpoint between response options. Probit regression coefficients were reported for 2 standard deviation difference in personality trait to make the small regression coefficients easier to read (i.e. the standardized personality scores were divided by two). The analysis of personality and self-reported health that allowed reporting heterogeneity (i.e. severity thresholds varying by personality traits) was carried out with the parametric approach (King et al., 2004), which is based on a compound hierarchical ordered probit (CHOPIT) model. This analysis was fitted with the anchors package of R software (Wand et al., 2011). For the analysis of personality and health vignette ratings, the data were transformed into long format so that each health vignette was a person-observation nested within the participant; robust estimator was used to estimate standard errors, and a categorical indicator of the health vignette type was included as an additional covariate. All the health problems items were skewed to the right, but this was not a problem for the ordered probit regression analysis.

Unless otherwise noted, all models included gender, age, and all the five personality traits as predictors. In HRS, the CHOPIT models were adjusted with three health vignettes that corresponded to the self-rated health domain. In WLS, each participant provided eight ratings for four vignettes of the total sixteen possible ratings of eight vignettes. The health vignettes varied in their severity, so some participants rated less and others more severe vignettes. In order to combine all participants in the same analysis, the eight missing values of the four vignettes for each participant were first imputed using stochastic regression imputation (i.e. multiple imputation with only single imputation). The imputation was carried out with all the health vignette ratings, self-rated health domains, personality traits, gender, and age as the predictors. The imputed data made it possible to use all the four health vignette ratings to adjust for reporting heterogeneity in the corresponding self-rated health. In the CHOPIT models, the vignettes were entered as separate variables (i.e. not as sum scores) in the model. The study was not preregistered.

## Results

Supplemental Table 1 shows the descriptive statistics. Table 1 shows the associations between personality traits and health vignette ratings. In WLS, higher conscientiousness, emotional stability, extraversion, and agreeableness were associated with more severe ratings of the vignettes, but these associations were not observed systematically in HRS. The magnitudes of the associations were modest. As shown in Supplemental Tables 2 and 3, the highest average correlation with health vignette ratings was *r* = 0.08 for emotional stability in HRS and *r* = 0.05 for agreeableness in WLS. Supplemental Tables 4 to 8 show how the personality traits were associated with varying severity thresholds that were used in the CHOPIT models to adjust for reporting heterogeneity. Overall, there were few, if any, systematic patterns in the associations between personality and rating thresholds.

**Table 1.** Personality traits predicting ratings of health vignettes.

Health and Retirement Study (*n* = 3950)						
Health problem	E	S	A	C	O	*R* ^2^
Sad/depressed	0.02 (0.03)	0.05 (0.03)	0.06 (0.03)	0.07 (0.04)	0.03 (0.04)	0.25
Sleeping	−0.06 (0.04)	0.05 (0.03)	**0.18 (0.04)**	**0.09 (0.04)**	0.01 (0.04)	0.01
Memory	**0.09 (0.03)**	**−0.06 (0.03)**	0.00 (0.03)	0.02 (0.03)	−0.01 (0.03)	0.22
Movement	0.01 (0.04)	−0.02 (0.03)	**0.09 (0.03)**	0.06 (0.03)	0.02 (0.03)	0.16
Shortness of breath	−0.03 (0.04)	0.02 (0.03)	0.06 (0.03)	**0.09 (0.03)**	**0.08 (0.04)**	0.15
Pain	**0.08 (0.03)**	−0.04 (0.03)	**0.08 (0.03)**	−0.06 (0.03)	−0.02 (0.03)	0.25
Wisconsin Longitudinal Study (*n* = 8664)						
Health problem	E	S	A	C	O	*R* ^2^
Sad/depressed	**0.05 (0.02)**	**0.15 (0.02)**	**0.09 (0.02)**	**0.10 (0.02)**	**0.08 (0.02)**	0.23
Worry/anxiety	**0.10 (0.02)**	**0.12 (0.02)**	**0.10 (0.02)**	**0.10 (0.02)**	0.01 (.02)	0.19
Movement	**0.05 (0.02)**	0.01 (0.02)	**0.04 (0.02)**	**0.08 (0.02)**	**0.15 (0.02)**	0.14
Vigorous activities	−0.03 (0.02)	**0.08 (0.02)**	0.02 (0.02)	**0.05 (0.02)**	**0.16 (0.02)**	0.11

Values are probit coefficients of 10 separate ordered probit regression models (6 in HRS, 4 in WLS) associated with 2 standard deviation difference in personality trait. Statistically significant coefficients are printed with bold font. Standard errors are in parenthesis.

E: extraversion; S: emotional stability; A: agreeableness; C: conscientiousness; O: openness to experience; *R*^2^: pseudo-*R*-squared of explained variability.

Table 2 reports the associations between personality traits and self-rated health outcomes with and without the adjustment for reporting heterogeneity based on the health vignette ratings. Higher conscientiousness, emotional stability, and extraversion were associated with less severe health problems in both cohorts. Higher agreeableness was associated with more severe health problems in HRS but not in WLS, while openness to experience had some both positive and negative associations in both cohorts. Table 3 illustrates the magnitude of associations as the predicted probabilities of reporting moderate, severe, or extreme health problems at low and high levels of personality traits (1 standard deviation below and above the mean).

**Table 2.** Personality traits predicting self-rated health problems.

Health and Retirement Study (*n* = 3950)						
Health problem	E	S	A	C	O	*R* ^2^
Sad/depressed	**−0.36 (0.05)**	**−0.87 (0.04)**	**0.22 (0.05)**	**−0.22 (0.04)**	0.05 (0.05)	0.08
+ Varying threshold	**−0.36 (0.06)**	**−0.83 (0.05)**	**0.21 (0.06)**	**−0.26 (0.05)**	0.04 (0.06)	
Sleeping	**−0.20 (0.05)**	**−0.51 (0.04)**	**0.09 (0.05)**	**−0.12 (0.04)**	0.03 (0.04)	0.03
+ Varying threshold	−0.06 (0.06)	**−0.54 (0.05)**	−0.07 (0.06)	**−0.27 (0.06)**	0.01 (0.06)	
Memory	**−0.17 (0.05)**	**−0.48 (0.04)**	**0.16 (0.05)**	**−0.43 (0.04)**	**−0.10 (0.05)**	0.06
+ Varying threshold^†^	**−0.28 (0.06)**	**−0.42 (0.04)**	**0.19 (0.06)**	**−0.38 (0.05)**	−0.02 (0.05)	
Movement	**−0.30 (0.05)**	**−0.40 (0.04)**	**0.20 (0.05)**	**−0.29 (0.04)**	0.06 (0.05)	0.04
+ Varying threshold	**−0.31 (0.06)**	**−0.35 (0.04)**	**0.18 (0.05)**	**−0.28 (0.05)**	0.07 (0.05)	
Shortness of breath	**−0.29 (0.05)**	**−0.38 (0.04)**	**0.12 (0.05)**	**−0.34 (0.05)**	**0.14 (0.05)**	0.04
+ Varying threshold	**−0.26 (0.07)**	**−0.36 (0.05)**	0.10 (0.07)	**−0.42 (**0.**06)**	0.10 (0.06)	
Pain	**−0.24 (0.05)**	**−0.44 (0.04)**	**0.25 (0.05)**	**−0.21 (0.04)**	0.05 (0.04)	0.03
+ Varying threshold^‡^	**−0.32 (0.05)**	**−0.41 (0.04)**	**0.19 (0.05)**	**−0.16 (0.05)**	0.08 (0.05)	
Average change (%)*	−4	6	7	−1	29	
Wisconsin Longitudinal Study (*n* = 8664)						
Health problem	E	S	A	C	O	*R* ^2^
Sad/depressed	**−0.30 (0.03)**	**−1.01 (0.03)**	**−0.08 (0.03)**	**−0.19 (0.03)**	**0.24 (0.03)**	0.11
+ Varying threshold	**−0.33 (0.04)**	**−1.13 (0.04)**	**−0.12 (0.04)**	**−0.24 (0.04)**	**0.11 (0.04)**	
Worry/anxiety	**−0.17 (0.03)**	**−1.28 (0.03)**	0.03 (0.03)	**−0.08 (0.03)**	**0.18 (0.03)**	0.14
+ Varying threshold	**−0.27 (0.04)**	**−1.36 (0.04)**	−0.04 (0.04)	**−0.21 (0.04)**	**0.16 (0.04)**	
Movement	**−0.14 (0.03)**	**−0.28 (0.03)**	−0.04 (0.03)	**−0.27 (0.03)**	0.02 (0.03)	0.03
+ Varying threshold	**−0.17 (0.03)**	**−0.33 (0.03)**	−0.03 (0.03)	**−0.26 (0.03)**	**−0.15 (0.03)**	
Vigorous activities	**−0.19 (0.03)**	**−0.19 (0.03)**	−0.02 (0.03)	**−0.30 (0.03)**	**−0.11 (0.02)**	0.03
+ Varying threshold	**−0.18 (0.03)**	**−0.25 (0.03)**	−0.03 (0.03)	**−0.32 (0.03)**	**−0.23 (0.03)**	
Average change (%)*	−21	−17	−50	−10	33	

Values are probit coefficients of 20 ordered probit regression models (12 in HRS, 8 in WLS) associated with 2 standard deviation difference in personality trait. Statistically significant coefficients are printed with bold font. Standard errors are in parenthesis.

E: extraversion; S: emotional stability; A: agreeableness; C: conscientiousness; O: openness to experience; *R*^2^: pseudo-*R*-squared of explained variabiliity.

† Conscientiousness was estimated in a separate model due to convergence problems. ^‡^Agreeableness and Openness estimated in a separate model due to convergence problems. *Average change indicates the mean change (%) in the coefficients between model without and with varying thresholds, averaged across the health problems but excluding health problems in which the change was more than 100%.

**Table 3.** Model-predicted health problems by levels of personality traits.

Health and Retirement Study (*n* = 3950)						
Health problem	Personality score	E	S	A	C	O
Sad/depressed	Low	20.7	28.1	13.1	18.8	15.1
Sad/depressed	High	11.9	7.4	18.4	13.5	16.3
Sleeping	Low	43.2	49.1	37.4	41.7	38.6
Sleeping	High	35.6	29.6	40.9	37.2	39.8
Memory	Low	18.9	23.2	14.6	23.1	18
Memory	High	14.6	11.2	18.5	12.3	15.4
Movement	Low	23.5	24.8	16.3	23.8	18.2
Movement	High	15.4	14	21.8	15.7	19.9
Shortness of breath	Low	13.1	14	9.1	14.1	8.9
Shortness of breath	High	8	7.2	11.3	7.7	11.5
Pain	Low	44.1	47.8	34.4	43.9	38.2
Pain	High	35	31	43.9	35.7	40.3
Wisconsin Longitudinal Study (*n* = 8664)						
Health problem	Personality score	E	S	A	C	O
Sad/depressed	Low	7.2	13.4	5.8	6.5	4.1
Sad/depressed	High	3.9	1.7	4.9	4.4	6.8
Worry/anxiety	Low	10.5	24.2	8.8	9.7	7.7
Worry/anxiety	High	7.7	2.4	9.3	8.4	10.6
Movement	Low	14.3	15.9	13.2	15.8	12.5
Movement	High	11.3	10	12.3	10.1	13
Vigorous activities	Low	57.6	57.6	54.2	59.8	56.1
Vigorous activities	High	50.2	50.2	53.5	47.9	51.7

Values are predicted probabilities of reporting moderate, severe, or extreme health problem by levels of personality trait (low = 1 standard deviation below the mean, high = 1 standard deviation above the mean).

E: extraversion; S: emotional stability; A: agreeableness; C: conscientiousness; O: openness to experience.

The associations did not change substantially when the probit models were fitted with CHOPIT that allowed the severity rating thresholds to vary as a function of personality (Table 2). To summarize the differences between models without and with varying thresholds, we first determined the percentage difference between their coefficients (e.g. in HRS, the association of emotional stability with the health problem of sad/depressed attenuated from −0.87 to −0.83, a 5% attenuation). This was done for all health problems, and the changes in coefficients were then averaged across all the health problems to get the average effect of varying thresholds on the associations (excluding those health problems for which the difference in coefficients was more than 100% because these appeared to be outliers, four associations in total). The coefficients of emotional stability, for example, were attenuated by an average of 6% in HRS but were strengthened by an average of 10% in WLS (when excluding the outlier symptom of worry/anxiety). For conscientiousness, there was no overall change in HRS (excluding sleeping problems) because the associations were attenuated for some health problems but strengthened for others. In WLS, the associations of conscientiousness were attenuated by an average of 10% (excluding worry/anxiety).

Three sensitivity analyses were fitted. First, to test whether the imputation procedure in WLS might have produced different results from a complete-case analysis, the CHOPIT models were fitted without data imputation and separately in each of the six subsamples who were assigned different pairs of vignettes (e.g. vignette 1 and 2; vignette 1 and 3; etc.). The subsample estimates were then combined using fixed-effect meta-analysis. The conclusions were essentially the same as with the main analysis done with data imputation (Supplemental Figures 1–5). Second, each personality trait was examined separately instead of being included in the model together with other personality traits (Supplemental Table 9). When modeled independently, agreeableness and openness to experience were both associated with fewer health problems, and not more health problems as in the mutually adjusted models (Table 2), suggesting that the positive associations were likely to be spurious and due to the overlap between different personality traits.

Third, the associations between personality traits and health vignette ratings (Table 1) were further adjusted for educational level (1 = less than high school, 2 = high school, 3 = more than high school) and cognitive ability (assessed with the Henmon–Nelson test in high school in WLS, and in the 2006 study wave in HRS with word recall (immediate and delayed) and mental state test) to examine whether the interpretation of health vignettes depended on the participant’s literacy level. Higher education and higher cognitive ability were associated with more severe ratings of most, but not all, vignettes (Supplemental Table 10). Yet their adjustments made very little difference to the associations between personality and vignette ratings (Supplemental Table 11). This suggests that the personality associations were not confounded by differences in education or cognitive ability that might influence how the participants are able to interpret the hypothetical vignettes.

## Discussion

The current analysis used fictional health vignettes to assess how strongly personality traits of the Five Factor Model were associated with severity ratings of fictional health conditions, and whether the differences in severity thresholds explained any of the associations between personality and self-rated health problems. Associations between personality and vignette ratings were modest (average correlations around *r* ≈ 0.05), the severity thresholds did not vary systematically with personality traits, and so the associations between personality and self-rated health outcomes did not change substantially when adjusted for the varying severity thresholds.

Personality traits might be associated with self-rated health problems because personality traits influence how people rate their health problems (Aschwanden et al., 2020). If this was the case, the associations between personality and health would be mostly about perceptions of health concerns rather than actual health concerns. The current results suggest that reporting heterogeneity associated with personality differences—measured by the associations between personality and spacings of health severity ratings—may not be a major source of bias in the study of personality and self-reported health.

Other methods besides health vignettes may be relevant when examining variability in health ratings. For example, the concept of “vague quantifiers” is relevant when considering how people may interpret expressions such as “very much,” “frequently,” or “severe” (Wright et al., 1994). Within-person scoring (ipsatization) and item response theory are some relevant statistical methods for evaluating individual variability in severity responses. The validity of self-reports might also be examined by comparing self-reported personality with personality scores derived from “behavioral residue” or “digital footprints,” such as social media use (Costello et al., 2021) or with ratings of informants, such as friends (Olino and Klein, 2015). The vignette approach is particularly useful for health research because it also provides insight into how individuals differ in their severity ratings of hypothetical health conditions. However, it should be noted that the specific health problems included in the vignettes of HRS and WLS have been selected somewhat arbitrarily to cover some of the most common health problems. The health problems do not follow any theoretical or conceptual model of health, and other health problems could have included, for which the results might have been different.

Personality has been associated with how people respond to different life adversities (Kendler et al., 2003). It therefore seems plausible that personality would also influence how people rate hypothetical health vignettes. However, the correlations between personality traits and vignette ratings turned out to be surprisingly small: the largest average correlation across all vignettes was *r* = 0.08 with emotional stability in HRS and *r* = 0.05 with agreeableness in WLS. The rating thresholds did not increase or decrease systematically with levels of personality traits. Thus, personality appears to be only weakly related to individual differences in rating severity of hypothetical health problems.

Most of the associations between personality traits and health problems were quite similar in HRS and WLS, with higher emotional stability, higher conscientiousness, and higher extraversion being associated with better health. Agreeableness was associated with poorer health in HRS, and openness to experience with poorer mental health but fewer problems with vigorous activities in WLS. But when these traits were examined independently of the other personality traits, they were both associated with fewer health problems in HRS and WLS (Supplemental Table 9). It thus seems that the positive associations were due to overlap between personality traits. Given that agreeableness and openness to experience have not been strongly associated with chronic diseases (Jokela, 2018) or symptoms of depression (Hakulinen et al., 2015), the cohort-specific positive associations should be interpreted with caution. There were also some differences between HRS and WLS in the association between personality traits and vignette ratings (Table 1), which might reflect methodological differences in sample characteristics (e.g. educational level), personality measures, or random variation (especially because the associations were modest in size).

### Limitations

Some limitations need to be considered. Health vignettes are not a perfect method to remove methodological confounds from self-reported health (Grol-Prokopczyk et al., 2015; Rice et al., 2011). In particular, the method relies on the assumptions of *vignette equivalence* and *response consistency* (King et al., 2004). Vignette equivalence refers to the assumption that the participants perceive the vignette to represent the same absolute level of health on a latent health scale. If personality is associated with differing interpretations of the absolute health level described in the vignette, then the effectiveness of the health vignettes is weakened; different participants no longer evaluate the same health state. Participants with personal experience with the health problem being rated might rate these vignettes differently than those without the personal experience, which could confound the method as well (e.g. individuals with low emotionality rating other people’s mental health problems as more severe because their own experiences with mental health problems).

Response consistency, in turn, is the assumption that the participants rate themselves and the vignette characters using the same criteria. If, for instance, individuals with high conscientiousness hold their own health to a different standard than they hold other people’s health, the ability of health vignettes to adjust for reporting heterogeneity is weakened. It is difficult to estimate how strong violations of these assumptions would have been required to alter the conclusions of the present analysis (Grol-Prokopczyk et al., 2015; van Soest and Vonkova, 2014). To further examine the validity of response consistency, it might be relevant to ask participants to rate hypothetical health problems that they themselves might have; this could be used to compare ratings of others versus self.

Another limitation is that both samples included mostly older adults from the United States in one time period (2003–2008), which may limit the generalizability of the findings to other populations. For example, the association between personality and self-rated health may change with age (Duberstein et al., 2003) or over time. Other characteristics might also moderate the associations between personality and health, and the role of differential reporting styles in these associations, including various psychosocial resources that determine how stressful the health conditions are for the respondent. Future studies should also examine whether facet-level analysis would be more informative than the higher-order trait level used in the current analysis. The Cronbach alphas of some of the sum scores of health vignette ratings were low, but the sum scores were not used in the main analysis of anchoring where each vignette rating was included as a separate variable. Some of the personality trait scales also had modest alphas of less than 0.7, which might have weakened the associations due to measurement error.

## Conclusions

Within the limitations of the vignette method (Grol-Prokopczyk et al., 2015), the present findings suggest that associations between personality and self-reported health problems may not be substantially confounded by reporting heterogeneity, that is, individual differences that arise from individuals having different severity thresholds for rating the severity of health problems. Before drawing definitive conclusions, studies with other methods to mitigate methodological confounds caused by self-reports are needed to support or refute the current findings. For instance, it would be useful to combine objective and self-reported measures to examine whether personality traits are differently related to functional limitations that are caused by objectively measured health problems (King et al., 2004). Such study designs would help us to better understand how personality is related not only to the development of illnesses but also to how different individuals are able to cope with the limitations of the illnesses.

## Supplemental Material

sj-docx-1-hpq-10.1177_13591053241285960 – Supplemental material for Reporting heterogeneity in the associations between personality and health problems: Anchoring self-reports with health vignettes Supplemental material, sj-docx-1-hpq-10.1177_13591053241285960 for Reporting heterogeneity in the associations between personality and health problems: Anchoring self-reports with health vignettes by Markus Jokela in Journal of Health Psychology
