# Supplementary material for: Reporting heterogeneity in the associations between personality and health problems: Anchoring self-reports with health vignettes
Source: J Health Psychol. 2024 Oct 6;30(11):3064–75. doi: 10.1177/13591053241285960 (PMC12433522; doi:10.1177/13591053241285960)
Supplement: sj-docx-1-hpq-10.1177_13591053241285960 – Supplemental material for Reporting heterogeneity in the associations between personality and health problems: Anchoring self-reports with health vignettes [file sj-docx-1-hpq-10.1177_13591053241285960.docx]

**Reporting heterogeneity in associations between personality and health problems: Anchoring self-reports with health vignettes**

Online Supplementary Material

**Vignettes rated for different health problems in the**

**Health and Retirement Study (HRS)**

**Pain**

Paul has a headache once a month that is relieved after taking a pill.

During the headache he can carry on with his day-to-day affairs.

Henry has pain that radiates down his right arm and wrist during his day at work. This is slightly relieved in the evenings when he is no longer working on his computer.

Charles has pain in his knees, elbows, wrists and fingers, and the pain is present almost all the time. Although medication helps, he feels uncomfortable when moving around, holding and lifting things.

**Sleep**

Maria takes about two hours every night to fall asleep. She wakes up once or twice a night feeling panicked and takes more than one hour to fall asleep again.

Karen wakes up almost once every hour during the night. When he wakes

up in the night, it takes around 15 minutes for her to go back to sleep. In

the morning she does not feel well-rested.

Alice falls asleep easily at night, but two nights a week she wakes up in the

middle of the night and cannot go back to sleep for the rest of the night.

**Sad/Depressed**

Maria feels nervous and anxious. She worries and thinks negatively about the future, but feels better in the company of people or when doing something that really interests her. When she is alone she tends to feel useless and empty.

Karen enjoys her work and social activities and is generally satisfied with her life. She gets depressed every 3 weeks for a day or two and loses interest in what she usually enjoys but is able to carry on with her day-to-day activities.

Anna feels depressed most of the time. She weeps frequently and feels hopeless about the future. She feels that she has become a burden on others and that she would be better dead.

**Shortness of breath**

Henry has been a heavy smoker for 30 years and wakes up with a

cough every morning. He gets short of breath even while resting and

does not leave the house anymore. He often needs to be put on oxygen.

Mark has no problems with walking slowly. He gets out of breath easily when climbing uphill for 20 meters or a flight of stairs.

Paul suffers from respiratory infections about once every year. He is short of breath 3 or 4 times a week and had to be admitted in hospital twice in the past month with a bad cough that required treatment with antibiotics.

**Movement**

Tom has a lot of swelling in his legs due to his health condition. He has to make an effort to walk around his home as his legs feel heavy.

Kevin does not exercise. He cannot climb stairs or do other physical activities because he is obese. He is able to carry the groceries and do some light household work.

Rob is able to walk distances of up to 200 metres without any problems but feels tired after walking one kilometre or climbing more than one flight of stairs. He has no problems with day-to-day activities, such as carrying food from the market.

**Memory**

Lisa can concentrate while watching TV, reading a magazine or playing a game of cards or chess. Once a week she forgets where her keys or glasses are, but

finds them within five minutes.

Sue is keen to learn new recipes but finds that she often makes mistakes and has to reread several times before she is able to do them properly.

Eve cannot concentrate for more than 15 minutes and has difficulty paying attention to what is being said to her. Whenever she starts a task, she never manages to finish it and often forgets what she was doing. She is able to learn the names of people she meets.

**Vignettes rated for different health problems in the**

**Wisconsin Longitudinal Study (WLS)**

**Movement and vigorous activities**

Imagine that the person described is the same age that you are. [Richard/Carol] is able to walk distances of up to 1/8 mile without any problems but feels tired after walking 1/2 mile or climbing up more than one flight of stairs. [He/she] has no problems with day-to-day physical activities, such as carrying food from the market.

Imagine that the person described is the same age that you are. [Robert/Mary] does not exercise. [He/she] cannot climb stairs or do other physical activities because [he/she] is obese. [He/she] is able to carry the groceries and do some light household work.

Imagine that the person described is the same age that you are. [Robert/Mary/Richard/Carol] has a lot of swelling in [his/her] legs due to [his/her] health condition. [He/she] has to make an effort to walk around [his/her] home as [his/her] legs feel heavy.

Imagine that the person described is the same age that you are. [Robert/Mary/Richard/Carol] is able to move [his/her] arms and legs, but requires assistance in standing up from a chair or walking around the house. Any bending is painful and lifting is impossible.

**Sad/depressed and anxiety/worry**

Imagine that the person described is the same age that you are. [James/Judith] enjoys [his/her] work and social activities and is generally satisfied with [his/her] life. [He/she] usually gets depressed every 3 weeks for a day or two and loses interest in what [he/she] usually enjoys but is able to carry on with [his/her] day-to-day activities.

Imagine that the person described is the same age that you are. [John/Barbara] worries about [his/her] health. [He/she] gets depressed once a week for a day or two, thinking about what could go wrong and all the illnesses [he/she] could get, but is able to come out of this mood if [he/she] concentrates on something else.

Imagine that the person described is the same age that you are. [James/Judith/John/Barbara] feels nervous and anxious. [He/she] worries and thinks negatively about the future, but feels better in the company of people or when doing something that really interests [him/her]. When [he/she] is alone [he/she] tends to feel useless and empty.

Imagine that the person described is the same age that you are. [James/Judith/John/Barbara] feels depressed most of the time. [He/she] weeps frequently and feels hopeless about the future. [He/she] feels that [he/she] has become a burden on others and that [he/she] would be better dead.

| **Supplementary Table 1.** Descripitive statistics | | | |  |
| --- | --- | --- | --- | --- |
|  | **HRS** | | **WLS** | |
|  | Mean (SD) | Range | Mean (SD) | Range |
| **Personality** |  |  |  |  |
| Extraversion | 2.21 (0.54) | 0–3 | 3.77 (0.86) | 1–6 |
| Emotional stability | 1.69 (0.69) | 0–3 | 3.97 (0.90) | 1–6 |
| Agreeableness | 2.55 (0.46) | 0–3 | 4.76 (0.70) | 1–6 |
| Conscientiousness | 2.45 (0.44) | 0–3 | 4.76 (0.69) | 1–6 |
| Openness to experience | 1.97 (0.53) | 0–3 | 3.57 (0.75) | 1–6 |
| **Self-reported health problems** | |  |  |  |
| Sad/Depressed | 1.77 (0.87) | 1–5 |  |  |
| Sleep | 2.28 (0.96) | 1–5 |  |  |
| Memory | 1.87 (0.78) | 1–5 |  |  |
| Movement | 1.76 (0.92) | 1–5 |  |  |
| Shortness of breath | 1.47 (0.79) | 1–5 |  |  |
| Pain | 2.37 (0.88) | 1–5 |  |  |
| Sad/Depressed |  |  | 1.46 (0.70) | 1–5 |
| Worry/Anxiety |  |  | 1.69 (0.77) | 1–5 |
| Movement |  |  | 1.54 (0.82) | 1–5 |
| Vigorous activities |  |  | 2.75 (1.43) | 1–5 |
| **Sociodemographic factors** | |  |  |  |
| Age | 65.1 (10.4) | 30–97 | 64.2 (4.2) | 34–87 |
| Gender (Women) | 60.7 (2,396) |  | 52.3 (4,528) |  |
| Race/Ethnicity* |  |  |  |  |
| White | 79.9 (3,156) |  | – |  |
| Black | 10.5 (413) |  | – |  |
| Hispanic | 7.4 (294) |  | – |  |
| Other | 2.2 (87) |  | – |  |
| Education |  |  |  |  |
| Less than high school | 13.2 (521) |  | 1.4 (117) |  |
| High school | 56.3 (2,224) |  | 68.7 (5,725) |  |
| More than high school | 30.5 (1,205) |  | 30.0 (2,498) |  |
| n | 3,950 |  | 8,664 |  |
| Note: * WLS includes no data on race/ethnicity, but the sample consists mostly of White, non-Hispanic participants. | | | |  |

| **Supplementary Table 2.** Correlations of personality and self-rated health with ratings of health vignettes in the Health and Retirement Study (n=3950). | | | | | | | | | | | |
| --- | --- | --- | --- | --- | --- | --- | --- | --- | --- | --- | --- |
|  | Personality | | | | | Self-rated health | | | | | |
|  | E | S | A | C | O | DEP | SLP | MEM | MOB | BRE | PAIN |
| Extraversion (E) |  |  |  |  |  |  |  |  |  |  |  |
| Emotional stability (S) | .22 |  |  |  |  |  |  |  |  |  |  |
| Agreeableness (A) | .55 | .14 |  |  |  |  |  |  |  |  |  |
| Conscientiousness (C) | .40 | .20 | .42 |  |  |  |  |  |  |  |  |
| Openness (O) | .52 | .23 | .38 | .46 |  |  |  |  |  |  |  |
| DEP | -.18 | -.38 | -.04 | -.15 | -.13 |  |  |  |  |  |  |
| SLP | -.12 | -.25 | -.04 | -.10 | -.10 | .37 |  |  |  |  |  |
| MEM | -.17 | -.23 | -.09 | -.24 | -.18 | .40 | .32 |  |  |  |  |
| MOB | -.16 | -.18 | -.05 | -.17 | -.11 | .34 | .39 | .38 |  |  |  |
| BRE | -.15 | -.16 | -.08 | -.17 | -.09 | .34 | .30 | .35 | .41 |  |  |
| PAIN | -.11 | -.21 | .00 | -.12 | -.08 | .35 | .41 | .34 | .66 | .35 |  |
| Vignette 1: DEP | .04 | .05 | .08 | .11 | .07 | -.06 | -.01 | -.06 | -.07 | -.05 | -.02 |
| Vignette 2: DEP | .06 | .03 | .07 | .07 | .07 | .02 | .02 | .01 | -.01 | .00 | .02 |
| Vignette 3: DEP | .04 | .01 | -.01 | -.01 | .01 | .03 | .06 | .03 | .06 | .04 | .06 |
| Vignette 1: SLP | .04 | .04 | .09 | .08 | .05 | -.02 | .05 | -.03 | -.06 | -.02 | .00 |
| Vignette 2: SLP | .05 | .00 | .10 | .08 | .05 | .01 | .06 | -.01 | -.01 | -.02 | .02 |
| Vignette 3: SLP | .04 | .04 | .09 | .08 | .05 | -.02 | .05 | -.03 | -.06 | -.02 | .00 |
| Vignette 1: MEM | .03 | -.05 | -.02 | -.05 | -.04 | .07 | .04 | .13 | .09 | .08 | .06 |
| Vignette 2: MEM | .03 | -.01 | .01 | -.01 | .01 | .08 | .09 | .12 | .11 | .09 | .10 |
| Vignette 3: MEM | .05 | .04 | .05 | .09 | .06 | -.02 | .04 | -.04 | .01 | -.01 | .03 |
| Vignette 1: MOB | .04 | .00 | .08 | .06 | .02 | -.01 | .06 | -.01 | .02 | -.01 | .03 |
| Vignette 2: MOB | .06 | .04 | .06 | .09 | .07 | -.03 | .01 | -.05 | -.04 | -.06 | -.02 |
| Vignette 3: MOB | .02 | -.03 | .00 | -.02 | .01 | .06 | .06 | .08 | .06 | .07 | .06 |
| Vignette 1: BRE | .02 | .02 | .00 | .02 | .05 | .02 | .05 | .02 | .05 | .05 | .07 |
| Vignette 2: BRE | .05 | .02 | .06 | .06 | .07 | -.02 | .04 | -.04 | -.01 | -.04 | .01 |
| Vignette 3: BRE | .04 | .02 | .08 | .11 | .06 | -.06 | .01 | -.06 | -.05 | -.04 | -.04 |
| Vignette 1: PAIN | .00 | -.04 | -.05 | -.09 | -.03 | .10 | .05 | .14 | .09 | .11 | .10 |
| Vignette 2: PAIN | .04 | .01 | .08 | .03 | .04 | .03 | .03 | .01 | -.01 | .02 | .04 |
| Vignette 3: PAIN | .05 | .00 | .07 | .04 | .02 | .02 | .06 | .01 | .01 | .02 | .03 |
| Note: Values are correlation coefficients. All values larger than \|.02\| are statistically significant. N=8664. DEP = Sad/Depressed, SLP=Sleeping, MEM=Memory/Concentration, MOB=Mobility, BRE=Shortness of breath, PAIN=Pain. | | | | | | | | | | | |

| **Supplementary Table 3.** Correlations of personality and self-rated health with ratings of health vignettes in the Wisconsin Longitudinal Study (n=8664). | | | | | | | | | |
| --- | --- | --- | --- | --- | --- | --- | --- | --- | --- |
|  | Personality | | | | | Self-rated health | | | |
|  | E | S | A | C | O | DEP | WOR | MOB | VIG |
| Extraversion (E) |  |  |  |  |  |  |  |  |  |
| Emotional stability (S) | .24 |  |  |  |  |  |  |  |  |
| Agreeableness (A) | .21 | .31 |  |  |  |  |  |  |  |
| Conscientiousness (C) | .26 | .32 | .36 |  |  |  |  |  |  |
| Openness (O) | .30 | .29 | .11 | .15 |  |  |  |  |  |
| Sad/Depressed | -.18 | -.40 | -.14 | -.20 | -.06 |  |  |  |  |
| Worry/Anxiety | -.16 | -.49 | -.11 | -.17 | -.09 | .66 |  |  |  |
| Mobility | -.11 | -.17 | -.07 | -.15 | -.06 | .28 | .27 |  |  |
| Vigorous activity | -.15 | -.18 | -.07 | -.19 | -.12 | .25 | .27 | .58 |  |
| Vignette 1: DEP | .08 | .11 | .05 | .08 | .03 | -.09 | -.08 | -.05 | -.05 |
| Vignette 2: DEP | .05 | .15 | .06 | .08 | .08 | -.11 | -.11 | -.03 | -.01 |
| Vignette 3: DEP | .08 | .13 | .11 | .11 | .10 | -.05 | -.04 | -.02 | .00 |
| Vignette 4: DEP | .06 | .07 | .08 | .09 | .08 | -.06 | -.05 | -.04 | .00 |
| Vignette 1: WOR | .07 | .04 | .05 | .08 | -.03 | -.04 | .00 | -.02 | -.04 |
| Vignette 2: WOR | .07 | .14 | .06 | .08 | .07 | -.09 | -.08 | -.01 | .02 |
| Vignette 3: WOR | .08 | .11 | .12 | .11 | .09 | -.04 | -.02 | -.02 | .01 |
| Vignette 4: WOR | .10 | .09 | .09 | .10 | .06 | -.06 | -.04 | -.02 | -.01 |
| Vignette 1: MOB | .08 | .08 | .01 | .04 | .12 | -.06 | -.05 | -.05 | -.12 |
| Vignette 2: MOB | .06 | .03 | .06 | .09 | .09 | .00 | -.01 | -.04 | -.06 |
| Vignette 3: MOB | .05 | .03 | .03 | .03 | .06 | -.02 | -.02 | -.03 | .01 |
| Vignette 4: MOB | .04 | .06 | .06 | .07 | .08 | .02 | .03 | -.01 | .01 |
| Vignette 1: VIG | .01 | .06 | .01 | .01 | .08 | .00 | -.01 | .01 | .11 |
| Vignette 2: VIG | .04 | .04 | .05 | .06 | .07 | .00 | .00 | .03 | .10 |
| Vignette 3: VIG | .02 | .04 | .03 | .04 | .06 | .00 | .01 | .00 | .13 |
| Vignette 4: VIG | .02 | .08 | .06 | .06 | .09 | -.01 | .01 | .02 | .11 |
| Note: Values are correlation coefficients. All values larger than \|.03\| are statistically significant. N=3950. DEP=Sad/Depressed, WOR=Worry/Anxiety, MOB=Mobility, VIG=Vigorous activities | | | | | | | | | |

| **Supplementary Table 4.** Varying thresholds of the CHOPIT model associated with extraversion | | | | |
| --- | --- | --- | --- | --- |
|  | Health and Retirement Study (n=3950) | | | |
| Vignette | None/Mild | Mild/Moderate | Moderate/Severe | Severe/Extreme |
| Sad/Depressed | 0.05 (0.04) | -0.12 (0.04) | 0.05 (0.03) | -0.08 (0.05) |
| Sleeping | 0.16 (0.07) | -0.01 (0.05) | -0.05 (0.04) | -0.10 (0.04) |
| Memory | -0.12 (0.04) | 0.03 (0.04) | 0.03 (0.04) | -0.01 (0.06) |
| Movement | -0.01 (0.04) | 0.01 (0.03) | -0.03 (0.03) | 0.02 (0.04) |
| Shortness of breath | 0.03 (0.05) | -0.03 (0.04) | 0.03 (0.04) | -0.04 (0.04) |
| Pain | -0.07 (0.03) | 0.01 (0.03) | -0.04 (0.03) | 0.02 (0.04) |
|  |  |  |  |  |
|  | Wisconsin Longitudinal Study (n=8664) | | | |
| Vignette | None/Mild | Mild/Moderate | Moderate/Severe | Severe/Extreme |
| Sad/Depressed | -0.02 (0.03) | -0.03 (0.02) | -0.01 (0.02) | 0.00 (0.03) |
| Worry/Anxiety | -0.09 (0.03) | -0.01 (0.03) | -0.01 (0.02) | -0.04 (0.03) |
| Movement | -0.03 (0.02) | 0.00 (0.02) | -0.02 (0.02) | 0.00 (0.02) |
| Vigorous activities | 0.00 (0.03) | 0.00 (0.02) | 0.03 (0.02) | -0.02 (0.01) |
| Note: Values are health threshold deviations associated with personality (2 standard deviation difference) in the probit models estimating the associations between personality and health problem, fitted separately for each health problem. Standard errors are in parenthesis. The threshold deviations are estimated based on the participants' responses to the health vignettes. Positive coefficients indicate that the personality trait was associated with a higher threshold of rating the vignette as more severe. | | | | |

| **Supplementary Table 5.** Varying thresholds of the CHOPIT model associated with emotional stability | | | | |
| --- | --- | --- | --- | --- |
|  | Health and Retirement Study (n=3950) | | | |
| Vignette | None/Mild | Mild/Moderate | Moderate/Severe | Severe/Extreme |
| Sad/Depressed | 0.07 (0.04) | -0.09 (0.03) | -0.03 (0.02) | 0.05 (0.04) |
| Sleeping | -0.04 (0.05) | 0.02 (0.04) | -0.02 (0.03) | 0.04 (0.03) |
| Memory | 0.09 (0.03) | -0.07 (0.03) | -0.01 (0.03) | 0.04 (0.05) |
| Movement | 0.07 (0.03) | -0.06 (0.03) | 0.00 (0.02) | 0.04 (0.03) |
| Shortness of breath | 0.03 (0.04) | -0.06 (0.03) | 0.02 (0.03) | 0.02 (0.03) |
| Pain | 0.03 (0.03) | 0.01 (0.03) | -0.01 (0.03) | 0.06 (0.04) |
|  |  |  |  |  |
|  | Wisconsin Longitudinal Study (n=8664) | | | |
| Vignette | None/Mild | Mild/Moderate | Moderate/Severe | Severe/Extreme |
| Sad/Depressed | -0.09 (0.03) | -0.10 (0.03) | -0.02 (0.02) | 0.05 (0.03) |
| Worry/Anxiety | -0.05 (0.03) | -0.12 (0.03) | -0.05 (0.02) | 0.07 (0.03) |
| Movement | -0.05 (0.02) | 0.01 (0.02) | 0.01 (0.02) | 0.07 (0.02) |
| Vigorous activities | -0.09 (0.03) | 0.04 (0.02) | -0.02 (0.02) | 0.04 (0.01) |
| Note: Values are health threshold deviations associated with personality (2 standard deviation difference) in the probit models estimating the assocaitions between personality and health problem, fitted separately for each health problem. Standard errors are in parenthesis. The threshold deviations are estimated based on the participants' responses to the health vignettes. Positive coefficients indicate that the personality trait was associated with a higher threshold of rating the vignette as more severe. | | | | |

| **Supplementary Table 6.** Varying thresholds of the CHOPIT model associated with agreeableness | | | | |
| --- | --- | --- | --- | --- |
|  | Health and Retirement Study (n=3950) | | | |
| Vignette | None/Mild | Mild/Moderate | Moderate/Severe | Severe/Extreme |
| Sad/Depressed | -0.01 (0.04) | 0.02 (0.04) | -0.05 (0.03) | 0.00 (0.05) |
| Sleeping | -0.14 (0.06) | -0.04 (0.05) | -0.01 (0.04) | -0.03 (0.04) |
| Memory | 0.04 (0.04) | 0.01 (0.04) | -0.10 (0.04) | 0.05 (0.06) |
| Movement | 0.00 (0.03) | -0.05 (0.03) | 0.02 (0.03) | -0.05 (0.04) |
| Shortness of breath | -0.03 (0.05) | 0.04 (0.04) | -0.04 (0.03) | -0.01 (0.04) |
| Pain | -0.01 (0.03) | -0.08 (0.03) | 0.01 (0.03) | 0.00 (0.02) |
|  |  |  |  |  |
|  | Wisconsin Longitudinal Study (n=8664) | | | |
| Vignette | None/Mild | Mild/Moderate | Moderate/Severe | Severe/Extreme |
| Sad/Depressed | 0.05 (0.02) | 0.01 (0.03) | -0.14 (0.03) | 0.05 (0.03) |
| Worry/Anxiety | -0.01 (0.02) | 0.01 (0.03) | 0.03 (0.02) | -0.05 (0.02) |
| Movement | 0.03 (0.02) | 0.03 (0.02) | -0.03 (0.03) | 0.02 (0.02) |
| Vigorous activities | 0.00 (0.02) | 0.01 (0.01) | 0.00 (0.00) | 0.00 (0.00) |
| Note: Values are health threshold deviations associated with personality (2 standard deviation difference) in the probit models estimating the assocaitions between personality and health problem, fitted separately for each health problem. Standard errors are in parenthesis. The threshold deviations are estimated based on the participants' responses to the health vignettes. Positive coefficients indicate that the personality trait was associated with a higher threshold of rating the vignette as more severe. | | | | |

| **Supplementary Table 7.** Varying thresholds of the CHOPIT model associated with conscientiousness | | | | |
| --- | --- | --- | --- | --- |
|  | Health and Retirement Study (n=3950) | | | |
| Vignette | None/Mild | Mild/Moderate | Moderate/Severe | Severe/Extreme |
| Sad/Depressed | -0.07 (0.04) | 0.07 (0.04) | -0.04 (0.03) | 0.03 (0.05) |
| Sleeping | -0.16 (0.06) | 0.00 (0.05) | 0.03 (0.04) | 0.03 (0.04) |
| Memory | 0.12 (0.03) | -0.10 (0.03) | -0.14 (0.03) | -0.05 (0.05) |
| Movement | 0.05 (0.03) | -0.07 (0.03) | -0.03 (0.03) | -0.03 (0.04) |
| Shortness of breath | -0.09 (0.04) | 0.05 (0.04) | 0.00 (0.03) | -0.06 (0.04) |
| Pain | 0.12 (0.03) | -0.09 (0.03) | 0.00 (0.03) | -0.01 (0.04) |
|  |  |  |  |  |
|  | Wisconsin Longitudinal Study (n=8664) | | | |
| Vignette | None/Mild | Mild/Moderate | Moderate/Severe | Severe/Extreme |
| Sad/Depressed | -0.04 (0.03) | 0.00 (0.02) | 0.01 (0.02) | -0.06 (0.03) |
| Worry/Anxiety | -0.06 (0.03) | -0.02 (0.03) | 0.01 (0.02) | -0.04 (0.03) |
| Movement | 0.01 (0.02) | -0.02 (0.02) | -0.01 (0.02) | 0.01 (0.02) |
| Vigorous activities | 0.00 (0.02) | -0.03 (0.02) | 0.02 (0.02) | -0.01 (0.01) |
| Note: Values are health threshold deviations associated with personality (2 standard deviation difference) in the probit models estimating the assocaitions between personality and health problem, fitted separately for each health problem. Standard errors are in parenthesis. The threshold deviations are estimated based on the participants' responses to the health vignettes. Positive coefficients indicate that the personality trait was associated with a higher threshold of rating the vignette as more severe. | | | | |

| **Supplementary Table 8.** Varying thresholds of the CHOPIT model associated with openness to experience | | | | |
| --- | --- | --- | --- | --- |
|  | Health and Retirement Study (n=3950) | | | |
| Vignette | None/Mild | Mild/Moderate | Moderate/Severe | Severe/Extreme |
| Sad/Depressed | 0.01 (0.04) | -0.02 (0.04) | -0.03 (0.03) | 0.01 (0.05) |
| Sleeping | 0.04 (0.06) | -0.11 (0.05) | 0.02 (0.04) | 0.06 (0.04) |
| Memory | 0.12 (0.04) | -0.09 (0.04) | -0.10 (0.04) | 0.04 (0.06) |
| Movement | 0.02 (0.03) | -0.05 (0.03) | -0.01 (0.03) | 0.00 (0.04) |
| Shortness of breath | -0.04 (0.05) | 0.00 (0.04) | -0.03 (0.03) | -0.05 (0.04) |
| Pain | 0.04 (0.03) | -0.01 (0.03) | -0.03 (0.03) | 0.01 (0.04) |
|  |  |  |  |  |
|  | Wisconsin Longitudinal Study (n=8664) | | | |
| Vignette | None/Mild | Mild/Moderate | Moderate/Severe | Severe/Extreme |
| Sad/Depressed | -0.17 (0.03) | 0.09 (0.02) | 0.05 (0.02) | 0.01 (0.03) |
| Worry/Anxiety | -0.04 (0.03) | 0.04 (0.03) | -0.01 (0.02) | 0.01 (0.03) |
| Movement | -0.19 (0.02) | 0.04 (0.02) | 0.03 (0.02) | 0.03 (0.02) |
| Vigorous activities | -0.16 (0.03) | 0.04 (0.02) | 0.00 (0.02) | 0.01 (0.01) |
| Note: Values are health threshold deviations associated with personality (2 standard deviation difference) in the probit models estimating the assocaitions between personality and health problem, fitted separately for each health problem. Standard errors are in parenthesis. The threshold deviations are estimated based on the participants' responses to the health vignettes. Positive coefficients indicate that the personality trait was associated with a higher threshold of rating the vignette as more severe. | | | | |

| **Supplementary Table 9.** Personality traits predicting self-rated health problems, each trait examined separately | | | | | |
| --- | --- | --- | --- | --- | --- |
|  | Health and Retirement Study (n=3950) | | | | |
| **Health problem** | **E** | **S** | **A** | **C** | **O** |
| Sad/Depressed | -0.47 (0.04) | -0.93 (0.04) | -0.18 (0.04) | -0.40 (0.04) | -0.34 (0.04) |
| + Varying threshold | -0.50 (0.04) | -0.92 (0.04) | -0.21 (0.04) | -0.45 (0.04) | -0.35 (0.04) |
| Sleeping | -0.30 (0.03) | -0.56 (0.03) | -0.14 (0.04) | -0.26 (0.04) | -0.22 (0.04) |
| + Varying threshold | -0.34 (0.05) | -0.62 (0.05) | -0.31 (0.05) | -0.44 (0.05) | -0.30 (0.05) |
| Memory | -0.41 (0.04) | -0.59 (0.04) | -0.23 (0.04) | -0.58 (0.04) | -0.43 (0.04) |
| + Varying threshold | -0.44 (0.04) | – | -0.23 (0.04) | – | -0.39 (0.04) |
| Movement | -0.36 (0.04) | -0.48 (0.04) | -0.12 (0.04) | -0.38 (0.04) | -0.25 (0.04) |
| + Varying threshold | -0.37 (0.04) | -0.50 (0.04) | -0.14 (0.04) | -0.38 (0.04) | -0.24 (0.04) |
| Shortness of breath | -0.36 (0.04) | -0.46 (0.04) | -0.20 (0.04) | -0.42 (0.04) | -0.21 (0.04) |
| + Varying threshold | -0.41 (0.05) | -0.48 (0.05) | -0.27 (0.05) | -0.52 (0.05) | -0.28 (0.05) |
| Pain | -0.26 (0.03) | -0.48 (0.03) | -0.03 (0.04) | -0.28 (0.04) | -0.18 (0.04) |
| + Varying threshold | – | – | – | – | -0.17 (0.04) |
|  |  |  |  |  |  |
|  | Wisconsin Longitudinal Study (n=8664) | | | | |
| **Health problem** | **E** | **S** | **A** | **C** | **O** |
| Sad/Depressed | -0.47 (0.03) | -1.07 (0.03) | -0.46 (0.03) | -0.50 (0.03) | -0.15 (0.03) |
| + Varying threshold | -0.58 (0.03) | -1.26 (0.04) | -0.56 (0.03) | -0.62 (0.03) | -0.32 (0.03) |
| Worry/Anxiety | -0.38 (0.02) | -1.27 (0.03) | -0.38 (0.03) | -0.42 (0.02) | -0.21 (0.02) |
| + Varying threshold | -0.54 (0.03) | -1.45 (0.04) | -0.52 (0.03) | -0.60 (0.03) | -0.30 (0.03) |
| Movement | -0.28 (0.03) | -0.40 (0.03) | -0.26 (0.03) | -0.40 (0.03) | -0.14 (0.03) |
| + Varying threshold | -0.36 (0.03) | -0.50 (0.03) | -0.29 (0.03) | -0.43 (0.03) | -0.33 (0.03) |
| Vigorous activities | -0.34 (0.02) | -0.37 (0.02) | -0.24 (0.02) | -0.43 (0.02) | -0.26 (0.02) |
| + Varying threshold | -0.39 (0.03) | -0.47 (0.03) | -0.29 (0.03) | -0.48 (0.03) | -0.40 (0.03) |
|  |  |  |  |  |  |
| Note: Values are probit coefficients for 2 standard deviation personality trait difference in predicting health problems when each personality trait is examined separately (adjusted for age and sex). A dash (–) indicates that the CHOPIT model did not converge. E = Extraversion, S = Emotional stability, A = Agreeableness, C = Conscientiousness, O = Openness to Experience. † Conscientiousness was estimated in a separate model due to convergence problems. ‡ Agreeableness and Openness estimated in a separate model due to convergence problems. * Average change indicates the mean change (%) in the coefficients between model without and with varying thresholds, averaged across the health problems but excluding health problems in which the change was more than 100%. | | | | | |

| **Supplementary Table 10.** Associations of education and cognitive ability with severity ratings of health vignettes. | | |
| --- | --- | --- |
| **Health and Retirement Study (n=3950)** | |  |
| Vignette | Education | Cognitive ability |
| Sad/Depressed | **0.12 (0.05)** | 0.10 (0.06) |
| Sleeping | **0.24 (0.06)** | **0.16 (0.07)** |
| Memory | **0.10 (0.04)** | **-0.13 (0.05)** |
| Movement | **0.12 (0.05)** | 0.07 (0.06) |
| Shortness of breath | 0.06 (0.05) | **0.14 (0.06)** |
| Pain | 0.09 (0.05) | -0.06 (0.06) |
| **Wisconsin Longitudinal Study (n=8664)** | | |
| Vignette | Education | Cognitive ability |
| Sad/Depressed | **0.04 (0.02)** | **0.04 (0.02)** |
| Worry/Anxiety | 0.00 (0.02) | 0.01 (0.02) |
| Movement | **0.07 (0.02)** | **0.06 (0.02)** |
| Vigorous activities | **0.07 (0.02)** | **0.20 (0.02)** |
| Note: Values are probit coefficients of 10 separate ordered probit regression models (6 in HRS, 4 in WLS) associated with 2 standard deviation difference in education and cognitive ability. Statistically significant coefficients are printed with bold font. Standard errors are in parenthesis. | | |

| **Supplementary Table 11.** Personality traits predicting ratings of health vignettes. | | | | |  |
| --- | --- | --- | --- | --- | --- |
|  | Health and Retirement Study (n=1763) | | | | |
| Vignette | E | S | A | C | O |
| **Adjusted for gender and age** | |  |  |  |  |
| Sad/Depressed | 0.06 (0.05) | -0.02 (0.04) | -0.01 (0.05) | 0.12 (0.05) | 0.02 (0.05) |
| Sleeping | 0.07 (0.06) | 0.03 (0.05) | 0.06 (0.06) | 0.10 (0.06) | -0.03 (0.06) |
| Memory | 0.16 (0.05) | -0.08 (0.04) | -0.06 (0.05) | 0.07 (0.05) | -0.03 (0.05) |
| Movement | 0.06 (0.06) | -0.01 (0.04) | 0.00 (0.05) | 0.12 (0.05) | -0.01 (0.05) |
| Shortness of breath | 0.02 (0.05) | 0.00 (0.04) | -0.01 (0.05) | 0.15 (0.05) | 0.04 (0.05) |
| Pain | 0.12 (0.05) | -0.09 (0.04) | 0.04 (0.05) | -0.09 (0.05) | -0.03 (0.05) |
| **Further adjusted for education and cognitive ability** | | | |  |  |
| Sad/Depressed | 0.08 (0.05) | -0.03 (0.04) | 0.00 (0.05) | 0.11 (0.05) | -0.02 (0.06) |
| Sleeping | 0.11 (0.06) | 0.01 (0.05) | 0.09 (0.06) | 0.08 (0.06) | -0.10 (0.07) |
| Memory | 0.16 (0.05) | -0.08 (0.04) | -0.06 (0.05) | 0.08 (0.05) | -0.04 (0.05) |
| Movement | 0.07 (0.06) | -0.03 (0.04) | 0.01 (0.05) | 0.11 (0.05) | -0.05 (0.05) |
| Shortness of breath | 0.03 (0.05) | -0.01 (0.04) | 0.00 (0.05) | 0.13 (0.05) | 0.01 (0.06) |
| Pain | 0.13 (0.05) | -0.09 (0.04) | 0.05 (0.05) | -0.08 (0.05) | -0.05 (0.05) |
|  |  |  |  |  |  |
|  | Wisconsin Longitudinal Study (n=7889) | | | | |
| Vignette | E | S | A | C | O |
| **Adjusted for gender and age** | |  |  |  |  |
| Sad/Depressed | 0.03 (0.01) | 0.08 (0.01) | 0.04 (0.01) | 0.06 (0.01) | 0.03 (0.01) |
| Worry/Anxiety | 0.06 (0.01) | 0.06 (0.01) | 0.05 (0.01) | 0.06 (0.01) | 0.00 (0.01) |
| Movement | 0.03 (0.01) | 0.01 (0.01) | 0.02 (0.01) | 0.04 (0.01) | 0.07 (0.01) |
| Vigorous activities | -0.02 (0.01) | 0.04 (0.01) | 0.02 (0.01) | 0.03 (0.01) | 0.08 (0.01) |
| **Further adjusted for education and cognitive ability** | | | |  |  |
| Sad/Depressed | 0.04 (0.01) | 0.07 (0.01) | 0.05 (0.01) | 0.06 (0.01) | 0.02 (0.01) |
| Worry/Anxiety | 0.06 (0.01) | 0.06 (0.01) | 0.05 (0.01) | 0.06 (0.01) | 0.00 (0.01) |
| Movement | 0.03 (0.01) | 0.00 (0.01) | 0.02 (0.01) | 0.04 (0.01) | 0.05 (0.01) |
| Vigorous activities | -0.01 (0.01) | 0.03 (0.01) | 0.03 (0.01) | 0.03 (0.01) | 0.04 (0.01) |
| Note: Values are ordered probit coefficients for 2 standard deviation difference in personality trait, adjusted for only age and sex, and then further for education and cognitive ability. E = Extraversion, S = Emotional stability, A = Agreeableness, C = Conscientiousness, O = Openness to Experience | | | | | |

**Supplementary Figure 1.** CHOPIT regression models for extraversion in the Wisconsin Longitudinal Study fitted separately for each of the vignette pair combinations and then pooled together using fixed-effect meta-analysis. Values are probit coefficients (and 95% confidence intervals) associated with 2 standard deviation difference in personality trait.

**Supplementary Figure 2.** CHOPIT regression models for emotional stability in the Wisconsin Longitudinal Study fitted separately for each of the vignette pair combinations and then pooled together using fixed-effect meta-analysis. Values are probit coefficients (and 95% confidence intervals) associated with 2 standard deviation difference in personality trait.

**Supplementary Figure 3.** CHOPIT regression models for agreeableness in the Wisconsin Longitudinal Study fitted separately for each of the vignette pair combinations and then pooled together using fixed-effect meta-analysis. Values are probit coefficients (and 95% confidence intervals) associated with 2 standard deviation difference in personality trait.

**Supplementary Figure 4.** CHOPIT regression models for conscientiousness in the Wisconsin Longitudinal Study fitted separately for each of the vignette pair combinations and then pooled together using fixed-effect meta-analysis. Values are probit coefficients (and 95% confidence intervals) associated with 2 standard deviation difference in personality trait.

**Supplementary Figure 5.** CHOPIT regression models for openness to experience in the Wisconsin Longitudinal Study fitted separately for each of the vignette pair combinations and then pooled together using fixed-effect meta-analysis. Values are probit coefficients (and 95% confidence intervals) associated with 2 standard deviation difference in personality trait.

HRS Data Formation (Stata)

// DATA FORMATION

clear all

set maxvar 30000

use rndhrs_o

keep hhidpn radyear radmonth r*agey_b

merge 1:1 hhidpn using h06f2b.dta, nogen

merge 1:1 hhidpn using h08f1b.dta, nogen

gen fvar=1

//

* id

clonevar id=hhidpn

rename hhid ohhid

clonevar hhid=hhidn

* sex

recode gender (1=0 "0 Male") (2=1 "1 Female"), gen(sex)

* age

// personality at wave1 (2006), split sample 1

*codebook klb033*, compact

local y=2004

foreach w in k l {

local y=`y'+2

recode `w'lb033a-`w'lb033z (4=0) (3=1) (2=2) (1=3)

recode `w'lb033p `w'lb033t (0=3) (1=2) (2=3) (3=0)

// Extraversion

* Outgoing, Friendly, Lively, Active, Talkative

egen nm_e`y'=rowmiss(`w'lb033a `w'lb033e `w'lb033i `w'lb033s `w'lb033w)

egen e`y'=rowmean(`w'lb033a `w'lb033e `w'lb033i `w'lb033s `w'lb033w) if nm_e`y' <=1

// Neuroticism

* Moody, Worrying, Nervous, Calm

egen nm_n`y'=rowmiss(`w'lb033c `w'lb033g `w'lb033k `w'lb033p)

egen n`y'=rowmean(`w'lb033c `w'lb033g `w'lb033k `w'lb033p) if nm_n`y' <=1

// Agreeableness

* Helpful, Warm, Caring, Softhearted, Sympathetic

egen nm_a`y'=rowmiss(`w'lb033b `w'lb033f `w'lb033j `w'lb033o `w'lb033v)

egen a`y'=rowmean(`w'lb033b `w'lb033f `w'lb033j `w'lb033o `w'lb033v) if nm_a`y' <=1

// Conscientiousness

* Organized, Responsible, Hardworking, Careless + Thorough

egen nm_c`y'=rowmiss(`w'lb033d `w'lb033h `w'lb033m `w'lb033t `w'lb033z)

egen c`y'=rowmean(`w'lb033d `w'lb033h `w'lb033m `w'lb033t `w'lb033z) if nm_c`y' <=1

// Openness to Experience

* Creative, Imaginative, Intelligent, Curious, Broad-minded, Sophisticated, Adventurous

egen nm_o`y'=rowmiss(`w'lb033l `w'lb033n `w'lb033q `w'lb033r `w'lb033u `w'lb033x `w'lb033y)

egen o`y'=rowmean(`w'lb033l `w'lb033n `w'lb033q `w'lb033r `w'lb033u `w'lb033x `w'lb033y) if nm_o`y' <=1

}

// combining 2006 & 2008 split samples

forvalues i=2006(2)2008 {

gen pe`i'=1 if e`i'<. | n`i'<. | a`i'<. | c`i'<. | o`i'<.

}

foreach p in e n a c o {

gen `p'1=`p'2006

replace `p'1=`p'2008 if `p'1==.

}

gen age1=r8agey_b if pe2006<.

replace age1=r9agey_b if pe2008<. & age1==.

// vignettes

merge 1:1 hhidpn using DVS07A_R, nogen

merge 1:1 hhidpn using DVS07B_R, nogen

egen vig_pain1=rowmin(apain1 bpain3) // Paul, Karen

egen vig_pain2=rowmin(apain2 bpain2) // Henry, Maria

egen vig_pain3=rowmin(apain3 bpain1) // Charles, Alice

egen vig_move1=rowmin(amove1 bmove3) // Tom, Sue

egen vig_move2=rowmin(amove2 bmove2) // Kevin, Lisa

egen vig_move3=rowmin(amove3 bmove1) // Rob, Eve

egen vig_memory1=rowmin(amemory1 bmemory3) // Lisa, Kevin

egen vig_memory2=rowmin(amemory2 bmemory2) // Sue, Tom

egen vig_memory3=rowmin(amemory3 bmemory1) // Eve, Rob

egen vig_breath1=rowmin(abreath1 bbreath3) // Mark, Karen

egen vig_breath2=rowmin(abreath2 bbreath2) // Paul, Karen

egen vig_breath3=rowmin(abreath3 bbreath1) // Henry, Maria

egen vig_depress1=rowmin(adepress1 bdepress3) // Anna, Mark

egen vig_depress2=rowmin(adepress2 bdepress2) // Maria, Henry

egen vig_depress3=rowmin(adepress3 bdepress1) // Karen, Paul

egen vig_sleep1=rowmin(asleep1 bsleep3) // Maria, Henry

egen vig_sleep2=rowmin(asleep2 bsleep2) // Karen, Paul

egen vig_sleep3=rowmin(asleep1 bsleep3) // Alice, Charles

gen lvar=1

keep fvar-lvar

//

foreach v in depress breath move memory sleep pain {

egen self_`v'=rowmin(aself_`v' bself_`v')

}

summ n1

gen s1=3-n1

foreach p in e s a c o {

egen std_`p'1=std(`p'1)

replace std_`p'1=std_`p'1/2

}

saveold data_hrs_vignette, replace version(12)

// descriptive statistics

oprobit self_depress age1 sex std_e1 std_s1 std_a1 std_c1 std_o1 vig_pain1

summ e1 s1 a1 c1 o1 age1 if e(sample)

tab sex if e(sample)

summ self_depress self_sleep self_memory self_move self_breath self_pain if e(sample)

// table 3, HRS

matrix t3hrs=J(6,10,.)

matrix t3hrs_p=J(6,5,.)

local nrow 0

foreach u in depress sleep memory move breath pain {

oprobit self_`u' age1 sex std_e1 std_s1 std_a1 std_c1 std_o1

local ++nrow

local ncol 0

local ncolp 0

foreach p in e s a c o {

lincom std_`p'1

local ++ncol

local ++ncolp

matrix t3hrs[`nrow',`ncol']=r(estimate)

matrix t3hrs_p[`nrow',`ncolp']=r(p)

local ++ncol

matrix t3hrs[`nrow',`ncol']=r(se)

}

}

matrix list t3hrs, format(%3.2f)

matrix list t3hrs_p, format(%4.3f)

// Supplementary Table 3, HRS

matrix hrsp=J(12,5,.)

local nrow -1

foreach u in depress sleep memory move breath pain {

local nrow = `nrow' + 2

oprobit self_`u' age1 sex std_e1 std_s1 std_a1 std_c1 std_o1

local ncol 0

foreach p in std_e1 std_s1 std_a1 std_c1 std_o1 {

local ++ncol

local nrowl = `nrow'

local nrowh = `nrow' + 1

margins, at(`p'=(-0.5 0.5)) atmeans predict(outcome(3)) predict(outcome(4)) predict(outcome(5))

matrix r=r(b)

matrix hrsp[`nrowl',`ncol']=r[1,1]+r[1,3]+r[1,5]

matrix hrsp[`nrowh',`ncol']=r[1,2]+r[1,4]+r[1,6]

}

}

matrix list hrsp

// Supplementary Table 1

oprobit self_depress age1 sex std_e1 std_s1 std_a1 std_c1 std_o1 vig_pain1

pwcorr e1 s1 a1 c1 o1 self_depress self_sleep self_memory self_move self_breath self_pain vig_depress? vig_sleep? vig_memory? vig_move? vig_breath? vig_pain? if e(sample), sig

// table 2, HRS

reshape long vig_pain vig_sleep vig_move vig_memory vig_breath vig_depress, i(hhidpn) j(vignette)

matrix t2hrs=J(6,10,.)

matrix t2hrs_p=J(6,5,.)

local nrow 0

foreach u in depress sleep memory move breath pain {

oprobit vig_`u' age1 i.vignette sex std_e1-std_o1, robust cluster(hhidpn)

local ++nrow

local ncol 0

local ncolp 0

foreach p in e s a c o {

lincom std_`p'1

local ++ncol

local ++ncolp

matrix t2hrs[`nrow',`ncol']=r(estimate)

matrix t2hrs_p[`nrow',`ncolp']=r(p)

local ++ncol

matrix t2hrs[`nrow',`ncol']=r(se)

}

}

*

matrix list t2hrs, format(%3.2f)

matrix list t2hrs_p, format(%4.3f)

WLS, Data Formation (Stata)

// WLS Graduates

clear all

set maxvar 30000

use wls_pub_13_03.dta

*sex, age

gen fvar=1

recode sexrsp (1=0 "0 Male") (2=1 "1 Female") (-3/-2=.), gen(sex)

gen age2=ga003re

gen id=_n

// personality, wave2

codebook ih001rec-ih039rer, comp

recode ih001rec-ih039rer (-3/-2=.)

recode ih0??rer (6=1) (5=2) (4=3) (3=4) (2=5) (1=6)

recode ih004rer ih006rer ih007rer ih011rer ih012rer ih014rer ih021rer ih022rer ih024rer ih028rer ih030rer ih034rer ih035rer ih038rer (1=6) (2=5) (3=4) (4=3) (5=2) (6=1)

*extraversion

egen nmiss_e2=rowmiss(ih003rer ih004rer ih005rer ih006rer ih007rer ih008rer)

egen e2=rowmean(ih003rer ih004rer ih005rer ih006rer ih007rer ih008rer) if nmiss_e2<=2

alpha ih003rer ih004rer ih005rer ih006rer ih007rer ih008rer, item

*neuroticism

egen nmiss_n2=rowmiss(ih027rer ih028rer ih029rer ih030rer ih031rer)

egen n2=rowmean(ih027rer ih028rer ih029rer ih030rer ih031rer) if nmiss_n2<=2

alpha ih027rer ih028rer ih029rer ih030rer ih031rer, item

*agreeableness

egen nmiss_a2=rowmiss(ih011rer ih012rer ih013rer ih014rer ih015rer ih016rer)

egen a2=rowmean(ih011rer ih012rer ih013rer ih014rer ih015rer ih016rer) if nmiss_a2<=2

alpha ih011rer ih012rer ih013rer ih014rer ih015rer ih016rer, item

*conscientiousness

egen nmiss_c2=rowmiss(ih019rer ih020rer ih021rer ih022rer ih023rer ih024rer)

egen c2=rowmean(ih019rer ih020rer ih021rer ih022rer ih023rer ih024rer) if nmiss_c2<=2

alpha ih019rer ih020rer ih021rer ih022rer ih023rer ih024rer, item

*openess

egen nmiss_o2=rowmiss(ih034rer ih035rer ih036rer ih037rer ih038rer ih039rer)

egen o2=rowmean(ih034rer ih035rer ih036rer ih037rer ih038rer ih039rer) if nmiss_o2<=2

alpha ih034rer ih035rer ih036rer ih037rer ih038rer ih039rer, item

// vignettes

// self

gen self_move2 = ix3001re

gen self_vigor2 = ix3002re

gen self_depress2 = ix3003re

gen self_worry2 = ix3004re

// vignette

gen amove2 = ixma2rer

gen avigor2 = ixma3rer

gen bmove2 = ixmb2rer

gen bvigor2 = ixmb3rer

gen cmove2 = ixmc2rer

gen cvigor2 = ixmc3rer

gen dmove2 = ixmd2rer

gen dvigor2 = ixmd3rer

// vignette

gen adepress2 = ixaa2rer

gen aworry2 = ixaa3rer

gen bdepress2 = ixab2rer

gen bworry2 = ixab3rer

gen cdepress2 = ixac2rer

gen cworry2 = ixac3rer

gen ddepress2 = ixad2rer

gen dworry2 = ixad3rer

recode self_move2 - dworry2 (min/-1=.)

gen lvar=1

keep id fvar-lvar

save data_wlsg, replace

*************************************************************************

// WLS, Siblings

clear all

set maxvar 30000

use wls_pub_13_03.dta

gen fvar=1

recode ssbsex (1=0 "0 Male") (2=1 "1 Female") (-3/-2=.), gen(sex)

gen age2=ca003re

gen id=_n

// personality

*wave2

codebook dh001rec-dh039rer, comp

recode dh001rec-dh039rer (-3/-2=.)

recode dh0??rer (6=1) (5=2) (4=3) (3=4) (2=5) (1=6)

recode dh004rer dh006rer dh007rer dh011rer dh012rer dh014rer dh021rer dh022rer dh024rer dh028rer dh030rer dh034rer dh035rer dh038rer (1=6) (2=5) (3=4) (4=3) (5=2) (6=1)

*extraversion

egen nmiss_e2=rowmiss(dh003rer dh004rer dh005rer dh006rer dh007rer dh008rer)

egen e2=rowmean(dh003rer dh004rer dh005rer dh006rer dh007rer dh008rer) if nmiss_e2<=2

alpha dh003rer dh004rer dh005rer dh006rer dh007rer dh008rer, item

*neuroticism

egen nmiss_n2=rowmiss(dh027rer dh028rer dh029rer dh030rer dh031rer)

egen n2=rowmean(dh027rer dh028rer dh029rer dh030rer dh031rer) if nmiss_n2<=2

alpha dh027rer dh028rer dh029rer dh030rer dh031rer, item

*agreeableness

egen nmiss_a2=rowmiss(dh011rer dh012rer dh013rer dh014rer dh015rer dh016rer)

egen a2=rowmean(dh011rer dh012rer dh013rer dh014rer dh015rer dh016rer) if nmiss_a2<=2

alpha dh011rer dh012rer dh013rer dh014rer dh015rer dh016rer, item

*conscientiousness

egen nmiss_c2=rowmiss(dh019rer dh020rer dh021rer dh022rer dh023rer dh024rer)

egen c2=rowmean(dh019rer dh020rer dh021rer dh022rer dh023rer dh024rer) if nmiss_c2<=2

alpha dh019rer dh020rer dh021rer dh022rer dh023rer dh024rer, item

*openess

egen nmiss_o2=rowmiss(dh034rer dh035rer dh036rer dh037rer dh038rer dh039rer)

egen o2=rowmean(dh034rer dh035rer dh036rer dh037rer dh038rer dh039rer) if nmiss_o2<=2

alpha dh034rer dh035rer dh036rer dh037rer dh038rer dh039rer, item

// VIGNETTES

// self

gen self_move2 = dx3001re

gen self_vigor2 = dx3002re

gen self_depress2 = dx3003re

gen self_worry2 = dx3004re

// vignette

gen amove2 = dxma2rer

gen avigor2 = dxma3rer

gen bmove2 = dxmb2rer

gen bvigor2 = dxmb3rer

gen cmove2 = dxmc2rer

gen cvigor2 = dxmc3rer

gen dmove2 = dxmd2rer

gen dvigor2 = dxmd3rer

// vignette

gen adepress2 = dxaa2rer

gen aworry2 = dxaa3rer

gen bdepress2 = dxab2rer

gen bworry2 = dxab3rer

gen cdepress2 = dxac2rer

gen cworry2 = dxac3rer

gen ddepress2 = dxad2rer

gen dworry2 = dxad3rer

recode self_move2 - dworry2 (min/-1=.)

gen lvar=1

keep id fvar-lvar

save data_wlss, replace

*************************************************************************

clear

use data_wlsg

append using data_wlss, gen(sibling)

drop nmiss*

replace id=id+10500 if sibling==1

set seed 2344277

* impute

reg sex age2 self_move2 self_vigor2 self_depress2 self_worry2 e2 n2 a2 c2 o2

keep if e(sample)

mi set wide

mi register imputed amove2-dworry2

mi register regular sex age2 self_move2 self_vigor2 self_depress2 self_worry2 e2 n2 a2 c2 o2

mi impute chained (regress) amove2-bvigor2 adepress2-bworry2 (ologit) cmove2 cvigor2 cdepress2 cworry2 dmove2 dvigor2 ddepress2 dworry2 = sex age2 self_move2 self_vigor2 self_depress2 self_worry2 e2 n2 a2 c2 o2, replace add(1)

foreach v of varlist _1_amove2-_1_dworry2 {

replace `v'=round(`v')

}

recode _1_amove2-_1_dworry2 (-1/0=1) (6/7=5)

rename self_move2 move_self2

rename self_vigor vigor_self2

rename self_depress2 depress_self2

rename self_worry2 worry_self2

foreach v in move vigor depress worry {

rename _1_a`v'2 vig1_`v'

rename _1_b`v'2 vig2_`v'

rename _1_c`v'2 vig3_`v'

rename _1_d`v'2 vig4_`v'

}

recode vig* (-1/0=1) (6/7=5)

gen s2=7-n2

foreach p in e2 s2 a2 c2 o2 {

egen std_`p' = std(`p')

}

foreach p in e2 s2 a2 c2 o2 {

replace std_`p' = std_`p'/2

}

// ANALYSIS

mi unset

foreach v in move vigor depress worry {

rename a`v'2 ovig1_`v'

rename b`v'2 ovig2_`v'

rename c`v'2 ovig3_`v'

rename d`v'2 ovig4_`v'

}

*

saveold data_wls_vignette, version(12) replace

// Descriptive statistics

oprobit move_self2 age2 sex std_e2-std_o2

summ e2 s2 a2 c2 o2 age if e(sample)

tab sex if e(sample)

summ depress_self2 worry_self2 move_self2 vigor_self2 if e(sample)

// Table 3, WLS

matrix t3wls=J(4,10,.)

matrix t3wls_p=J(4,5,.)

local nrow 0

foreach u in depress worry move vigor {

oprobit `u'_self2 age2 sex std_e2-std_o2

local ++nrow

local ncol 0

local ncolp 0

foreach p in e s a c o {

lincom std_`p'2

local ++ncol

local ++ncolp

matrix t3wls[`nrow',`ncol']=r(estimate)

matrix t3wls_p[`nrow',`ncolp']=r(p)

local ++ncol

matrix t3wls[`nrow',`ncol']=r(se)

*

}

}

matrix list t3wls, format(%3.2f)

matrix list t3wls_p, format(%4.3f)

// Supplementary Table 3, WLS

matrix wlsp=J(8,5,.)

local nrow -1

foreach u in depress worry move vigor {

local nrow = `nrow' + 2

oprobit `u'_self2 age2 sex std_e2-std_o2

local ncol 0

foreach p in std_e2 std_s2 std_a2 std_c2 std_o2 {

local ++ncol

local nrowl = `nrow'

local nrowh = `nrow' + 1

margins, at(`p'=(-0.5 0.5)) atmeans predict(outcome(3)) predict(outcome(4)) predict(outcome(5))

matrix r=r(b)

matrix wlsp[`nrowl',`ncol']=r[1,1]+r[1,3]+r[1,5]

matrix wlsp[`nrowh',`ncol']=r[1,2]+r[1,4]+r[1,6]

}

}

matrix list wlsp

// Supplementary Table 2

oprobit move_self2 age2 sex std_e2-std_o2

pwcorr std_e2-std_o2 depress_self2 worry_self2 move_self2 vigor_self2 vig?_depress vig?_worry vig?_move vig?_vigor if e(sample), sig

// Table 2, WLS

// reshape to long

reshape long ovig@_move ovig@_vigor ovig@_depress ovig@_worry vig@_move vig@_vigor vig@_depress vig@_worry , i(id) j(vignette)

//

matrix t2wls=J(4,10,.)

matrix t2wls_p=J(4,5,.)

local nrow 0

foreach u in depress worry move vigor {

oprobit vig_`u' age2 i.vignette sex std_e2-std_o2, robust cluster(id)

local ++nrow

local ncol 0

local ncolp 0

foreach p in e s a c o {

lincom std_`p'2

local ++ncol

local ++ncolp

matrix t2wls[`nrow',`ncol']=r(estimate)

matrix t2wls_p[`nrow',`ncolp']=r(p)

local ++ncol

matrix t2wls[`nrow',`ncol']=r(se)

}

}

matrix list t2wls, format(%3.2f)

matrix list t2wls_p, format(%4.3f)

*

matrix list t3wls, format(%3.2f)

matrix list t3wls_p, format(%4.3f)

CHOPIT Analysis (R)

install.packages("anchors")

install.packages("hopit")

install.packages("foreign")

library(anchors)

library(hopit)

library(foreign)

hrs <- read.dta("data_hrs_vignette.dta")

wls <- read.dta("data_wls_vignette.dta")

hrs.depress <- list(self = self_depress ~ sex + age1 + std_e1 + std_s1 + std_a1 + std_c1 + std_o1,

vign = cbind(vig_depress1, vig_depress2, vig_depress3) ~ 1 ,

tau = ~ std_e1 + std_s1 + std_a1 + std_c1 + std_o1)

#

hrs.sleep <- list(self = self_sleep ~ sex + age1 + std_e1 + std_s1 + std_a1 + std_c1 + std_o1,

vign = cbind(vig_sleep1, vig_sleep2, vig_sleep3) ~ 1 ,

tau = ~ std_e1 + std_s1 + std_a1 + std_c1 + std_o1)

#

hrs.memory <- list(self = self_memory ~ sex + age1 + std_e1 + std_s1 + std_a1 + std_c1 + std_o1,

vign = cbind(vig_memory1, vig_memory2, vig_memory3) ~ 1 ,

tau = ~ std_e1 + std_s1 + std_a1 + std_o1)

#

hrs.memory.c1 <- list(self = self_memory ~ sex + age1 + std_e1 + std_s1 + std_a1 + std_c1 + std_o1,

vign = cbind(vig_memory1, vig_memory2, vig_memory3) ~ 1 ,

tau = ~ std_c1)

#

hrs.move <- list(self = self_move ~ sex + age1 + std_e1 + std_s1 + std_a1 + std_c1 + std_o1,

vign = cbind(vig_move1, vig_move2, vig_move3) ~ 1 ,

tau = ~ std_e1 + std_s1 + std_a1 + std_c1 + std_o1)

#

hrs.breath <- list(self = self_breath ~ sex + age1 + std_e1 + std_s1 + std_a1 + std_c1 + std_o1,

vign = cbind(vig_breath1, vig_breath2, vig_breath3) ~ 1 ,

tau = ~ std_e1 + std_s1 + std_a1 + std_c1 + std_o1)

#

hrs.pain <- list(self = self_pain ~ sex + age1 + std_e1 + std_s1 + std_a1 + std_c1 + std_o1,

vign = cbind(vig_pain1, vig_pain2, vig_pain3) ~ 1 ,

tau = ~ std_e1 + std_s1 + std_c1)

#

hrs.pain.a1.o1 <- list(self = self_pain ~ sex + age1 + std_e1 + std_s1 + std_a1 + std_c1 + std_o1,

vign = cbind(vig_pain1, vig_pain2, vig_pain3) ~ 1 ,

tau = ~ std_a1 + std_o1)

# Table 3, HRS

# Supplementary Tables 4 to 8

hrs.depress.chopit <- chopit(hrs.depress, data=hrs)

summary(hrs.depress.chopit)

hrs.sleep.chopit <- chopit(hrs.sleep, data=hrs)

summary(hrs.sleep.chopit)

hrs.memory.chopit <- chopit(hrs.memory, data=hrs)

summary(hrs.memory.chopit)

hrs.memory.chopit.c1 <- chopit(hrs.memory.c1, data=hrs)

summary(hrs.memory.chopit.c1)

hrs.move.chopit <- chopit(hrs.move, data=hrs)

summary(hrs.move.chopit)

hrs.breath.chopit <- chopit(hrs.breath, data=hrs)

summary(hrs.breath.chopit)

hrs.pain.chopit <- chopit(hrs.pain, data=hrs)

summary(hrs.pain.chopit)

hrs.pain.chopit.a1.o1 <- chopit(hrs.pain.a1.o1, data=hrs)

summary(hrs.pain.chopit.a1.o1)

#

wls.depress <- list(self = depress_self2 ~ sex + age2 + std_e2 + std_s2 + std_a2 + std_c2 + std_o2,

vign = cbind(vig1_depress, vig2_depress, vig3_depress, vig4_depress) ~ 1 ,

tau = ~ std_e2 + std_s2 + std_a2 + std_c2 + std_o2)

#

wls.worry <- list(self = worry_self2 ~ sex + age2 + std_e2 + std_s2 + std_a2 + std_c2 + std_o2,

vign = cbind(vig1_worry, vig2_worry, vig3_worry, vig4_worry) ~ 1 ,

tau = ~ std_e2 + std_s2 + std_a2 + std_c2 + std_o2)

#

wls.move <- list(self = move_self2 ~ sex + age2 + std_e2 + std_s2 + std_a2 + std_c2 + std_o2,

vign = cbind(vig1_move, vig2_move, vig3_move, vig4_move) ~ 1 ,

tau = ~ std_e2 + std_s2 + std_a2 + std_c2 + std_o2)

#

wls.vigor <- list(self = vigor_self2 ~ sex + age2 + std_e2 + std_s2 + std_a2 + std_c2 + std_o2,

vign = cbind(vig1_vigor, vig2_vigor, vig3_vigor, vig4_vigor) ~ 1 ,

tau = ~ std_e2 + std_s2 + std_a2 + std_c2 + std_o2)

# Table 3, WLS

# Supplementary Tables 4 to 8, WLS

wls.depress.chopit <- chopit(wls.depress, data=wls)

summary(wls.depress.chopit)

wls.worry.chopit <- chopit(wls.worry, data=wls)

summary(wls.worry.chopit)

wls.move.chopit <- chopit(wls.move, data=wls)

summary(wls.move.chopit)

wls.vigor.chopit <- chopit(wls.vigor, data=wls)

summary(wls.vigor.chopit)
